# Double-balloon endoscopy WAS effective in diagnosing small intestinal duplication: a case report

**DOI:** 10.1186/s40064-016-3256-4

**Published:** 2016-09-19

**Authors:** Yuji Nadatani, Toshio Watanabe, Takashi Sugawa, Shinpei Eguchi, Sunao Shimada, Koji Otani, Hirokazu Yamagami, Tetsuya Tanigawa, Masatsugu Shiba, Kazunari Tominaga, Yasuhiro Fujiwara, Tetsuo Arakawa

**Affiliations:** 1 Department of Gastroenterology, Osaka City University Graduate School of Medicine, 1-4-3 Asahimachi, Abeno-ku, Osaka City, Osaka 545-8585 Japan; 2 SAMURAI GI Research Centre, Osaka City University Graduate School of Medicine, 1-4-3 Asahimachi, Abeno-ku, Osaka City, 545-8585 Japan

**Keywords:** Double-balloon endoscopy, Ileal duplication, Small intestine

## Abstract

**Introduction:**

Ileal duplications are encountered infrequently in adults, because symptoms including abdominal pain, intussusception, hemorrhage, and perforation usually present in early childhood. In this report, we present an adult case of ileal duplication that was revealed by double-balloon endoscopy (DBE).

**Case description:**

A 73-year-old Japanese man presented with anemia and melena. Anal DBE detected the narrow opening of an extra lumen in the ileum about 100 cm proximal to the ileocecal valve. Enteroclysis via DBE showed a 5-cm-long ileal diverticulum-like structure at the mesenteric side of the ileum. No ectopic gastric mucosa was detected by technetium-99m pertechnetate scintigraphy. The final diagnosis was ileal duplication.

**Discussion and evaluation:**

This is the first report of tubular ileal duplication diagnosed by using DBE. The small intestinal duplication opening was not detected by using VCE and plane CT in this case, but was found by using DBE.

**Conclusions:**

The present case demonstrates that DBE was useful in the diagnosis of an adult small intestinal duplication that was not visualized by other modalities.

## Introduction

Enteric duplication is a rare congenital disease. It is mainly observed in the small intestine, although it can occur anywhere in the gastrointestinal tract. Ileal duplications are encountered infrequently in adults, because symptoms including abdominal pain, intussusception, hemorrhage, and perforation usually present in early childhood (Cheng et al. 2014; Furuya et al. 2012). Most adult enteric duplications are diagnosed incidentally in perioperative or autopsy cases because noninvasive conventional radiologic and endoscopic techniques provide limited information about small intestinal disease. We can now diagnose enteric duplication endoscopically because of recent advances in medical technology. In this report, we present an adult case of ileal duplication that was revealed by double-balloon endoscopy (DBE).

## Case description

A 73-year-old Japanese man whose only significant medical history included endoscopic submucosal dissection for early gastric cancer in 2013 and 2014 visited our hospital because of abdominal pain, anemia, and melena in June 2015. Esophagogastroduodenoscopy and colonoscopy revealed no bleeding sources other than the small intestine. Although his symptoms improved without treatment soon after visiting the hospital, he was admitted for further investigation of the cause of anemia and melena in February 2016.

**Fig. 1:**
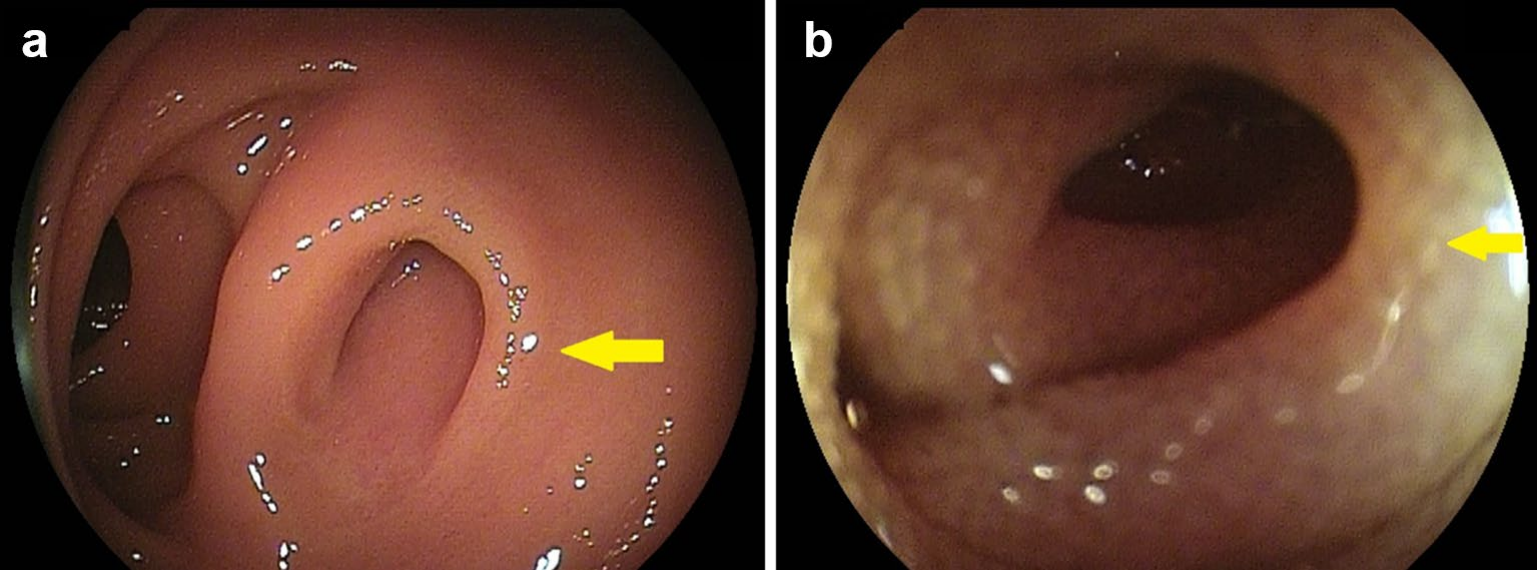
Endoscopic images of the ileal duplication. Double-balloon endoscopy (DBE) detected the narrow opening of an extra lumen in the ileum about 100 cm proximal to the ileocecal valve

**Fig. 2:**
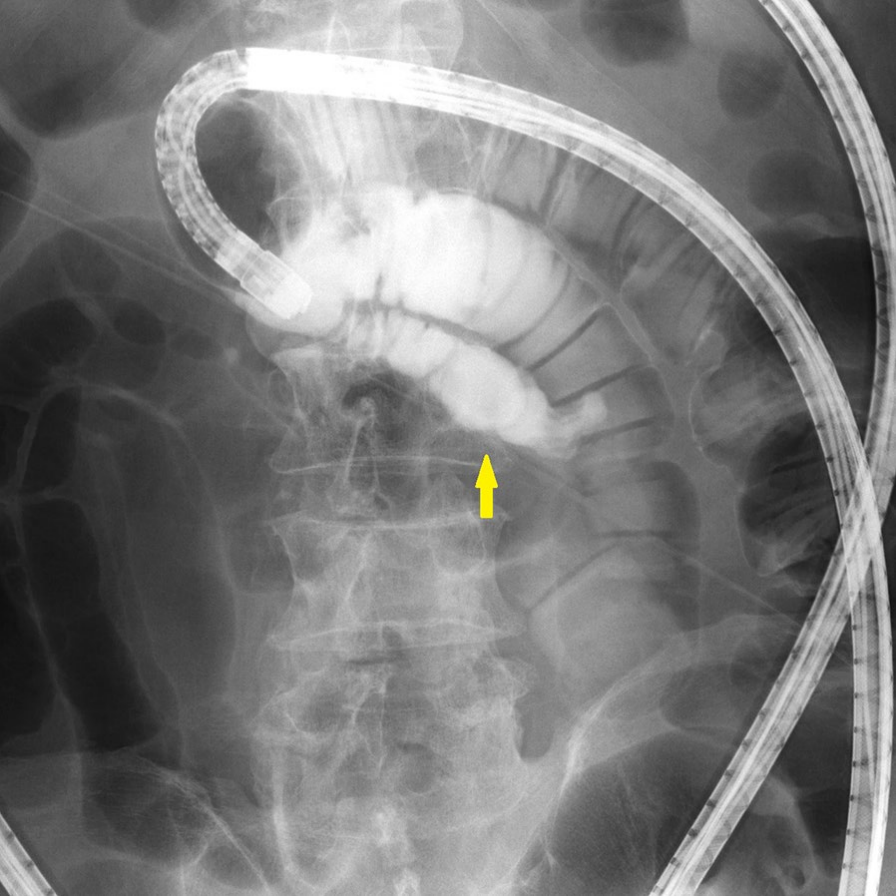
Enteroclysis image of ileal duplication. Enteroclysis via DBE using Gastrografin showed a 5-cm-long ileal diverticulum-like structure on the mesenteric side of the ileum

**Fig. 3:**
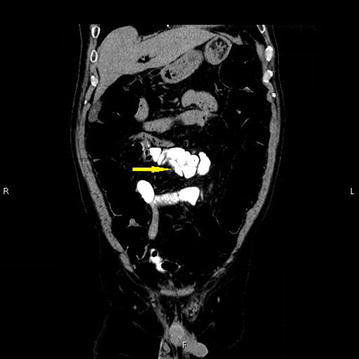
Abdominal CT enteroclysis image. CT enteroclysis was performed immediately after DBE enteroclysis in order to confirm the shape and size. There were no malignant findings in the duplication. The arrow shows the duplication

Physical examination was unremarkable. There was no abdominal tenderness. A complete blood count and laboratory data, including tumor marker levels, were within normal limits. Abdominal contrast-enhanced computed tomography (CT) was unremarkable. Video-capsule endoscopy (VCE) revealed some redness at the ileum. We performed oral and anal DBE in February 2016. Anal DBE showed the narrow opening of an extra lumen in the ileum about 100 cm proximal to the ileocecal valve (Fig. 1). The extra lumen was located on the mesenteric side of the ileum and had a blind end. The biopsy specimens obtained from the ileal duplication showed histologically normal small intestinal tissue. Enteroclysis via DBE using Gastrografin (diatrizoate meglumine and diatrizoate sodium solution) (Nihon Schering, Osaka, Japan), a water-soluble iodinated radiopaque contrast medium, showed a 5-cm-long ileal diverticulum-like structure at the mesenteric side of the ileum (Fig. 2). Abdominal CT enteroclysis was performed immediately after DBE enteroclysis in order to confirm the shape and size (Fig. 3). No ectopic gastric mucosa was detected by technetium-99m pertechnetate scintigraphy. The patient was discharged soon after these unremarkable examinations.

The final diagnosis was ileal duplication. We concluded that the abdominal pain, anemia, and melena were caused by the ileal duplication. We will perform surgical excision, including partial resection of the ileum.

## Discussion and evaluation

This is the first report of tubular ileal duplication diagnosed by using DBE. Enteric duplication can occur anywhere from the esophagus to the large intestine. It is a congenital anomaly commonly situated on the mesenteric side of the digestive tract and shares the same blood supply. Some enteric duplications, which are called enteric duplication cysts, don’t have openings. Enteric duplications are primarily discovered perioperatively because of severe bleeding, abdominal pain, perforation, or similar symptoms; mildly symptomatic or asymptomatic enteric duplications are rarely diagnosed. There are few reports of preoperative diagnosis, especially in adults. Holcomb et al. reviewed 77 enteric duplications and reported that about 70 % were found in children under the age of 2 years (Holcomb et al. 1989).

Some small intestinal duplications are diagnosed as a result of malignant change. The rate of malignant change of enteric duplications is very low, but we should keep in mind their potential for malignant transformation. Furuya et al. reported 9 cases of malignant change in ileal duplications in patients aged older than 15 years (Furuya et al. 2012). Although we confirmed the absence of malignant findings by CT, enteroclysis, and other testing, surgical resection is being planned. As scheduled in our case, the role of surgery should be considered in all symptomatic patients.

Although VCE is noninvasive and can completely evaluate the small intestine as well as detect small intestinal duplications (Yang et al. 2009), DBE is the first choice of diagnostic modality to assess a duplication. There are several reasons why we recommend DBE rather than VCE. First, capsule retention risk is high in small intestinal duplication patients because ulcerated or stenotic lesions are a well-known retention risk factor of VCE. Ervin et al. reported capsule retention for several weeks in a nearby circumferential ulcerated stenotic lesion in a small intestinal duplication. Second, a small intestinal duplication opening is detected more easily by DBE than VCE, because of the ability to observe details and perform enteroclysis via the DBE scope channel. Actually, the small intestinal duplication opening was not detected by using VCE in this case, but was found by using DBE. Therefore, we should choose DBE examination rather than VCE if we suspect a small intestinal duplication.

Previous studies suggested that intestinal duplications might contain ectopic gastric mucosa that may perforate and bleed, similar to a Meckel’s diverticulum (Holcomb et al. 1989; Niesche 1973; Collins 1972; Bhattacharya et al. 2013). Therefore, we should confirm whether ectopic gastric mucosa is present in an enteric duplication. In this case, we performed Meckel’s diverticulum scintigraphy and confirmed the absence of ectopic gastric mucosa.

## Conclusions

In conclusion, we encountered a case of small intestinal duplication diagnosed by DBE. DBE is the best modality for use in subjects presenting with symptoms that may indicate small intestinal duplication when other modalities fail to make a diagnosis, because we can directly detect the narrow opening and evaluate the shape using direct enteroclysis.
